# Efficacy of Second-Line Pemetrexed-Carboplatin in EGFR-Activating Mutation-Positive NSCLC: Does Exon 19 Deletion Differ from Exon 21 Mutation?

**DOI:** 10.1155/2017/8196434

**Published:** 2017-10-23

**Authors:** Amit Joshi, Vanita Noronha, Vijay M. Patil, Anuradha Chougule, Atanu Bhattacharjee, Rajiv Kumar, Supriya Goud, Sucheta More, Anant Ramaswamy, Ashay Karpe, Nikhil Pande, Arun Chandrasekharan, Alok Goel, Vikas Talreja, Abhishek Mahajan, Amit Janu, Nilendu Purandare, Kumar Prabhash

**Affiliations:** ^1^Department of Medical Oncology, Tata Memorial Hospital, Mumbai, India; ^2^Centre for Cancer Epidemiology, ACTREC, Tata Memorial Centre, Mumbai, India; ^3^Department of Pathology, Tata Memorial Hospital, Mumbai, India; ^4^Department of Radiology, Tata Memorial Hospital, Mumbai, India; ^5^Department of Nuclear Medicine, Tata Memorial Hospital, Mumbai, India

## Abstract

**Background:**

It is unknown whether the outcomes of second-line pemetrexed-carboplatin chemotherapy administered after progression on gefitinib are dependent on type of EGFR mutation present at baseline.

**Method:**

Adult non-small-cell lung cancer patients, with exon 19 deletion or exon 21 L858R mutation, who progressed on gefitinib and received pemetrexed-carboplatin chemotherapy were selected for this analysis.

**Result:**

55 patients received pemetrexed-carboplatin as second-line treatment. Response rates in evaluable patients were 39.3% in exon 19 patients (*n* = 28) and 33.3% in exon 21 patients (*n* = 15) (*p* = 0.752, Fisher's exact 2-sided *p* value). The median PFS in exon 19 and 21 cohorts was 5.900 months (95% CI: 4.274–7.526) and 4.767 months (95% CI: 1.374–8.159), respectively. The median overall survival in exon 19 patients was (11.8 months, 95% CI: 9.916–13.684 months) significantly better than that seen in exon 21 mutation patients (6.2 months, 95% CI: 4.215–8.118 months, *p* = 0.024) on univariate analysis; however, on multivariate analysis, this association was not confirmed (HR = 0.361, 95% CI: 0.090–1.439, *p* = 0.149).

**Conclusion:**

Exon 19 deletion has no impact on PFS and OS in EGFR-mutated patients treated with second-line pemetrexed-carboplatin.

## 1. Introduction

The treatment of EGFR exon 19-deleted and exon 21 L858R-substituted non-small-cell lung cancer (NSCLC) is through tyrosine kinase inhibitor (TKI) [1]. Reversible and irreversible tyrosine kinase inhibitors have proven their worth against platinum doublet chemotherapy agents in multiple studies [2–4]. In majority of these studies done across the globe, TKIs lead to an improvement in treatment-related outcomes. A common theme in each of these studies was the selection of NSCLC patients with classic EGFR-activating mutations. Classic EGFR-activating mutations consist of exon 19 deletion and exon 21 L858R substitution.

Till recently, these 2 mutations were clubbed together in all studies. However, data has emerged now that exon 19 is a biologically distinct subtype with a favourable prognosis [5]. The response rates, progression-free survival (PFS), and overall survival (OS) of exon 19 deletion patients treated with TKI are significantly better than those of exon 21 mutation patients. Commonly, these classic EGFR-activating mutated patients at progression are treated with platinum doublet chemotherapy. Pemetrexed is frequently the agent with platinum of choice. Whether the outcomes of second-line pemetrexed-carboplatin chemotherapy administered after progression on gefitinib are dependent on the type of EGFR mutation present at baseline is unknown.

We recently published a randomized study in EGFR-mutated NSCLC warranting palliative therapy comparing pemetrexed-carboplatin to gefitinib in first-line setting (Clinical Trials Registry-India: CTRI/2015/08/006113) [6]. We conducted a post hoc analysis on patients who progressed on gefitinib and received pemetrexed-carboplatin to address the above-mentioned question.

## 2. Methods

### 2.1. Patient Selection

Adult NSCLC patients, with exon 19 deletion or exon 21 L858R mutation, who progressed on gefitinib and received pemetrexed-platinum chemotherapy were selected for this analysis. The study was approved by the institutional ethics committee. All patients provided written informed consent and the study was conducted in accordance with the norms laid down by Declaration of Helsinki and good clinical practice guidelines.

### 2.2. Intervention

These patients were offered biopsy at progression. Patients who were willing were started on pemetrexed and carboplatin after the biopsy. Pemetrexed (500 mg/m^2^) in combination with carboplatin (AUC-5) was administered with supportive medications consisting of appropriate antiemetics, vitamin B12, folic acid, and dexamethasone. Patients underwent response assessment after 3rd cycle, after 6th cycle, and then every 2 months thereafter. The doublet regimen was administered for 6 cycles and if the subject had nonprogressive disease, they were shifted to pemetrexed maintenance. The maintenance was continued till the patient had either intolerable side effects or progressive disease. Patients were followed up till death.

### 2.3. Statistical Analysis

SPSS version 20 was used for analysis. Best response rate to second-line therapy was documented in accordance with RECIST version 1.1 and compared with Fisher's exact test. Progression-free survival was defined as time in months from date of start of second-line treatment to date of progression, date of change in treatment, or death from any cause, whichever occurred earlier. Overall survival was defined as time in months from date of start of second-line treatment to death from any cause. Patients who had not died at last follow-up were censored on 14 July 2016. Kaplan-Meier time to event analysis was used for estimation of PFS and OS. Log-rank test was used for comparison of PFS and OS between exon 19 deletion and exon 21 mutation patients. Cox regression analysis was used to estimate the hazard ratio with its 95% confidence interval. A *p* value of 0.05 or below was considered as significant.

## 3. Results

### 3.1. Baseline Details

55 patients received pemetrexed-carboplatin as second-line treatment. Exon 19 deletion was seen in 33 patients (60%) and exon 21 mutation was seen in 22 patients (40%). The median age was 55 years (35–76 years). There were 29 males (52.7%) and 26 females (47.3%). Fifteen patients (27.2%) had a history of previous smoking. The site of progression was intrathoracic site in 37 patients (71.2%), extrathoracic site in 5 patients (9.1%), and both sites in 13 patients (19.7%). The ECOG PS was 0-1 in 52 patients (94.5%) and 2 in 3 patients (4.5%). The distribution of baseline characteristics in accordance with the type of mutation is shown in Table 1.

### 3.2. Response Rate

The overall response rate was 29.1% (Table 2). There were no cases of complete response. Response rate in evaluable patients was 39.3% in exon 19 patients (*n* = 28) and 33.3% in exon 21 patients (*n* = 15) (*p* = 0.752, Fisher's exact 2-sided *p* value).

### 3.3. PFS

**Figure 1:**
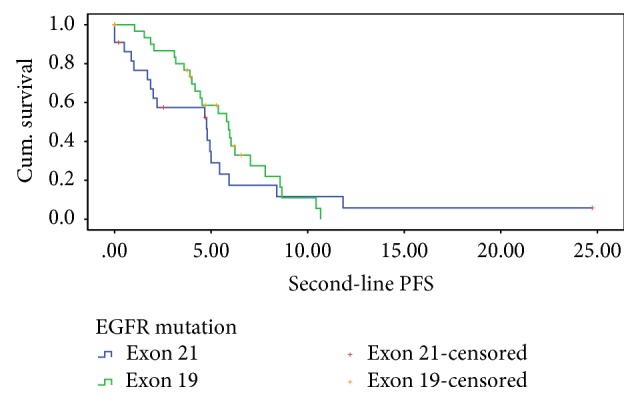
Estimated progression-free survival in the 2 cohorts.

At the time of data cutoff, 76.4% of the patients had progressed. The overall median PFS was 4.933 months (95% CI: 4.086–5.781). The median PFS in exon 19 and exon 21 cohorts was 5.900 months (95% CI: 4.274–7.526) and 4.767 months (95% CI: 1.374–8.159), respectively (Figure 1). There was a trend towards better PFS with exon 19 (*p* = 0.121, HR = 0.563, 95% CI: 0.272–1.164).  Table 3 provides the details of the results of Cox regression analysis.

### 3.4. OS

**Figure 2:**
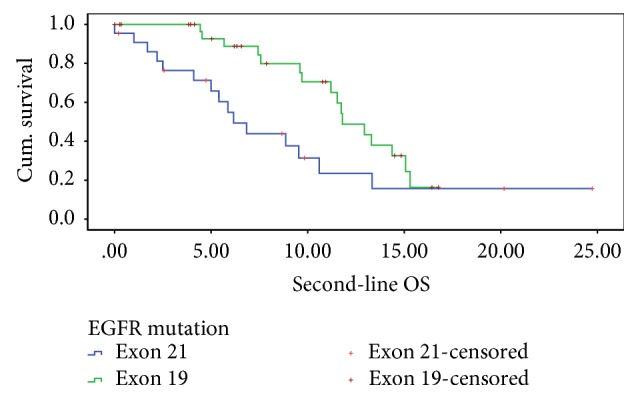
Estimated overall survival in the 2 cohorts.

At the time of data cutoff, 56.4% of the patients had died. The overall median survival was 11.2 months (95% CI: 8.677–13.723 months). The median overall survival in exon 19 deletion patients was (11.8 months, 95% CI: 9.916–13.684 months) significantly better than that seen in exon 21 mutation patients (6.2 months, 95% Cl: 4.215–8.118 months, *p* = 0.024) on log-rank test (Figure 2); however, after adjusting for treatment after failure on pemetrexed and carboplatin, there was no difference in the survival between the two subtypes.  Table 4 provides the details of the results of Cox regression analysis.

## 4. Discussion

EGFR mutation in lung cancer is a favourable biomarker. EGFR-mutated disease is associated with a longer overall survival than EGFR-non-mutated NSCLC when appropriately treated [7]. TKIs are considered as the current standard first line of treatment [1]. Tissue biopsy is recommended when patients progress on oral TKI as it helps in identifying epithelial mesenchymal transition and also helps to identify new mutations like T790M. The frequencies of development of this mutation are similar in the exon 19 and exon 21 cohorts. However, getting a tissue sample is difficult at recurrence because of multiple reasons. These can include patient's refusal, progression at a site inaccessible for biopsy, or patient's condition that might preclude such an invasive procedure. Second-line chemotherapy with pemetrexed platinum doublet is frequently administered when no actionable mutation is identified or if biopsy was not feasible.

In the last few years, the differential impact of exon 19 mutation over exon 21 mutation has been identified. Exon 19 patients when treated with TKI have a better response rate, progression-free survival, and overall survival than exon 21 mutation patients [5, 8, 9]. Recent evidence now recommends separate evaluation of these mutation patients in future studies. However, whether the difference in the outcomes of these mutations is seen in presence of oral TKI or whether administration of pemetrexed doublet would also have a differential impact is not known. In first-line setting, in a small study of 32 patients, there was no differential impact of pemetrexed seen [10]. Similar findings were reported by us too [11]. However, administration of pemetrexed in first-line setting in EGFR-mutated cancers is an unlikely scenario in the current era.

This study answers the question of differential outcomes in the presence of other mutation clones of exon 19 and exon 21. The study does suggest that there is no statistical difference in progression-free survival and overall survival associated with baseline presence of exon 19 deletion. The hazard ratio for mortality in presence of exon 19 mutation was 0.361, implicating a trend towards decrease in mortality associated with this mutation. The treatment after pemetrexed-carboplatin failure was received in higher proportion of patients with exon 19 deletion than those with exon 21 mutation. The reason for this is unclear. Probably the ECOG PS was more preserved in exon 19 deletion patients after failure on pemetrexed-carboplatin.

Multiple ways of resistance are suggested in literature for development of TKI resistance. T790M is one of them [12, 13]. In nearly half of the patients with gefitinib resistance, T790M mutation is seen [14]. Unfortunately, Osimertinib, a tyrosine kinase inhibitor active in T790M mutation, was not available in India during the study period. Hence, the biopsy of these patients was only used to rule out development of small-cell carcinoma. It would have been interesting to know whether the differential impact of type of mutation would have been seen in presence of T790M mutation.

The current study has its own limitations. The sample size was small, biopsies were not performed in all patients at progression, and it was a post hoc analysis. However, the data for the current analysis was mined from the prospective database of the Phase 3 study and therefore, although the analysis plan was retrospective, the data was collected prospectively.

## 5. Conclusion

Exon 19 deletion has no impact on PFS and OS in EGFR-mutated patients treated with second-line pemetrexed-carboplatin.

## Figures and Tables

**Table 1:** Baseline characteristics in the 2 cohorts.

Variable	Exon 19 (*n* = 33)	Exon 21 (*n* = 22)
Median age	51 (38–76)	57.55 (35–69)

Gender		
Male	19 (57.6%)	10 (68.6%)
Female	14 (33.4%)	12 (31.4%)

ECOG PS		
0-1	30 (90.9%)	22 (100%)
2	3 (9.1%)	—

Habits		
Ex-smoker	10 (30.3%)	5 (22.7%)

Brain metastasis	5 (33.4%)	5 (22.7%)

**Table 2:** Response to pemetrexed in the 2 cohorts.

Variable	Exon 19 (*n* = 33)	Exon 21 (*n* = 22)
CR	—	—
PR	11	5
SD	11	5
PD	6	5
Not evaluable	5	7

**Table 3:** Details of multivariate analysis for progression-free survival.

Variable	Hazard ratio	*p* value on Cox regression analysis
Elderly^*∗*^	1.710 (0.486–6.019)	0.430
ECOG PS	0.354 (0.98–1.288)	0.115
Smoking status	1.474 (0.671–3.239)	0.334
Brain metastasis	0.932 (0.416–2.090)	0.865
EGFR mutation type	0.563 (0.272–1.164)	0.121

^*∗*^Elderly was defined as age of 65 years or above.

**Table 4:** Details of multivariate analysis for overall survival.

Variable	Hazard ratio	*p* value on Cox regression analysis
Elderly^*∗*^	>100 (0.0–NA)	0.980

ECOG PS	0.321 (0.078–1.322)	0.116

Smoking status	1.709 (0.475–6.153)	0.412

Brain metastasis	0.844 (0.176–4.042)	0.832

EGFR mutation type	0.361 (0.090–1.439)	0.149

Treatment received after failure on pemetrexed-carboplatin	0.265 (0.060–1.163)	0.078

^*∗*^Elderly was defined as age of 65 years or above. NA: not applicable.
